# Differential timing of granule cell production during cerebellum development underlies generation of the foliation pattern

**DOI:** 10.1186/s13064-016-0072-z

**Published:** 2016-09-08

**Authors:** Emilie Legué, Jackie L. Gottshall, Edouard Jaumouillé, Alberto Roselló-Díez, Wei Shi, Luis Humberto Barraza, Senna Washington, Rachel L. Grant, Alexandra L. Joyner

**Affiliations:** 1 Developmental Biology Program, Memorial Sloan Kettering Cancer Center, New York, NY 10065 USA; 2 Neuroscience Program of Weill Cornell Graduate School of Medical Sciences, New York, USA; 3 Biochemistry, Cell and Molecular Biology Program of Weill Cornell Graduate School of Medical Sciences, New York, USA; 4 Present Address: Department of Pediatrics, Yale University School of Medicine, 333 Cedar Street, New Haven, CT 06520 USA

**Keywords:** Engrailed genes, *En2*, gcps, Lobules, Proliferation, Differentiation

## Abstract

**Background:**

The mouse cerebellum (Cb) has a remarkably complex foliated three-dimensional (3D) structure, but a stereotypical cytoarchitecture and local circuitry. Little is known of the cellular behaviors and genes that function during development to determine the foliation pattern. In the anteroposterior axis the mammalian cerebellum is divided by lobules with distinct sizes, and the foliation pattern differs along the mediolateral axis defining a medial vermis and two lateral hemispheres. In the vermis, lobules are further grouped into four anteroposterior zones (anterior, central, posterior and nodular zones) based on genetic criteria, and each has distinct lobules. Since each cerebellar afferent group projects to particular lobules and zones, it is critical to understand how the 3D structure of the Cb is acquired. During cerebellar development, the production of granule cells (gcs), the most numerous cell type in the brain, is required for foliation. We hypothesized that the timing of gc accumulation is different in the four vermal zones during development and contributes to the distinct lobule morphologies.

**Methods and Results:**

In order to test this idea, we used genetic inducible fate mapping to quantify accumulation of gcs in each lobule during the first two postnatal weeks in mice. The timing of gc production was found to be particular to each lobule, and delayed in the central zone lobules relative to the other zones. Quantification of gc proliferation and differentiation at three time-points in lobules representing different zones, revealed the delay involves a later onset of maximum differentiation and prolonged proliferation of gc progenitors in the central zone. Similar experiments in *Engrailed* mutants (*En1*^*−/+*^*;En2*^*−/−*^), which have a smaller Cb and altered foliation pattern preferentially outside the central zone, showed that gc production, proliferation and differentiation are altered such that the differences between zones are attenuated compared to wild-type mice.

**Conclusions:**

Our results reveal that gc production is differentially regulated in each zone of the cerebellar vermis, and our mutant analysis indicates that the dynamics of gc production plays a role in determining the 3D structure of the Cb.

**Electronic supplementary material:**

The online version of this article (doi:10.1186/s13064-016-0072-z) contains supplementary material, which is available to authorized users.

## Background

The cortex of the mammalian cerebellum (Cb) has a layered cytoarchitecture and the basic cellular processes that produce the layers are the same throughout the Cb [1, 2]. Contrasting with its homogenous cellular organization, the Cb displays a complex three-dimensional (3D) structure with the anteroposterior (a-p) axis being divided by fissures into lobules [3]. Lobules are grouped into four a-p zones based on specific gene expression patterns [4, 5]: the anterior (AZ, lobules 1–5), central (CZ, lobules 6–7), posterior (PZ, lobule 8 and anterior 9) and the nodular zones (NZ, posterior lobule 9 and 10). Foliation has two distinct patterns along the mediolateral (m-l) axis, defining a central vermis flanked by two hemispheres. The complex 3D organization of the Cb along two Cartesian axes parallels the organization of the afferents that project to the Cb [3]. Depending on their origin, afferents target specific sets of lobules, some primarily in either the vermis or the hemispheres, thus suggesting a modular organization of cerebellar functions [6, 7]. Therefore, the acquisition of the 3D structure of the Cb during development is a crucial step in the assembly of the long-range cerebellar circuitry.

The relatively simple cytoarchitecture of the Cb underlies its stereotyped organization of local neuronal circuitry. Granule cells (gcs) and sparsely distributed interneurons compose the inner most layer of the cerebellar cortex, the inner granule cell layer (IGL). The adjacent Purkinje cell layer (PcL) contains Pc cell bodies and Bergmann glia (Bg), and the outer most molecular layer (ML) has Bergmann glial fibers, Pc dendrites, gc axons (the parallel fibers) and interneurons. This cellular organization is identical throughout the cerebellar cortex, despite the diverse size and shape of lobules that house local circuits and the various long-range circuits in which they are integrated.

The Cb has two developmental progenitor zones [1, 8]. The ventricular zone produces the Pcs, Bg, interneurons and astrocytes [9], whereas the upper rhombic lip generates the projection neurons of the cerebellar nuclei and then the gcs. Proliferating gc precursors (gcps) first migrate over the surface of the cerebellar primordium to form the external granule cell layer (EGL) [10, 11]. Gcps proliferate in the outer layers of the EGL (oEGL); once they exit the cell cycle, gcs form an inner layer of the EGL (iEGL) where they extend horizontal processes and undergo tangential migration along the m-l axis. Subsequently, gcs extend a vertical process through the forming ML and the cell bodies migrate along Bg fibers and past the PcL to finally settle in the IGL [12–17]. The parallel fibers of newly differentiated gcs stack on top of the parallel fibers of previously differentiated gcs [18]. Thus the ML grows by addition of new gc axons from inside to outside. In contrast, the positions of gc bodies within the IGL are not correlated with the time of birth [19]. Unlike the neural precursors in the VZ, gcps in the EGL divide symmetrically to expand the progenitor pool and then to produce differentiated neurons. Quite remarkably, individual clones of gcps differentiate *en masse* over a short time period (<3 days) and the position of their parallel fibers in the ML indicates the time when the cells of a clone differentiated while the cell bodies are scattered in the IGL [19, 20].

The process of foliation (by which the Cb acquires lobules) is initiated at late embryonic stages in mouse (E17.5) and proceeds through the first two post-natal weeks when gc production is maximal. Fissure formation is initiated by sequential changes in cell behaviors at specific locations that become the base of each fissure, termed “anchoring centers” [21]. All anchoring centers are not initiated at the same time; instead they form in a reproducible sequence [20]. The shapes and sizes of lobules are diverse but the overall pattern of foliation is highly conserved within a species and throughout mammals [22, 23], suggesting the positioning of the anchoring centers is under genetic control. Indeed, in mouse mutants in which the timing and positioning of the anchoring centers are altered, the pattern of foliation is changed (e.g., [21, 24, 25]). However, it is not well understood how the diversity in lobule size and shape is produced during Cb development.

Recently, we discovered that clones of gcps initiated around birth are confined to the EGL region between two anchoring centers and that the size and geometry of clones are different in short and long lobules [20]. We proposed that the compartmentalization of gcs into lobules allows for differential regulation of cell behaviors in each lobule. In order to gain further insight into the origin of the diversity of lobule shapes and sizes, we used genetic inducible fate mapping [26] to test whether differential timing of gc production (increase in gc number) underlies the acquisition of cerebellar 3D structure. Indeed we found that the peak of gc production is most similar for lobules within a zone, and that the CZ is generally delayed. Evaluation of gc proliferation and differentiation showed that the relative delay of gc production in the CZ involves delayed differentiation of gcps compared to the AZ and prolonged proliferation compared to the AZ and NZ. Similar analyses in mouse *En1*^*−/+*^;*En2*^*−/−*^ mutants, which have a smaller Cb and preferential loss of AZ and PZ lobules [27], showed that the differences in gc production, proliferation and differentiation seen between zones of wild-type (WT) mice are diminished in mutants, correlating with the preferential loss of AZ and PZ lobules.

## Methods

### Mouse lines

The *Atoh1-CreER*^*T2*^ [10], *Tau*^*lox-STOP-lox-mGFP*-*IRES-NLSlacZ*^ [28], *En1*^*hd*^ [29] and *En2*^*ntd*^ [30] mouse lines were maintained on an outbred Swiss-Webster background and genotyped as previously described. All animal studies were performed under an approved Institutional Animal Care and Use Committee mouse protocol according to MSKCC institutional guidelines. Noon of the day that a vaginal plug was detected was designated as embryonic day (E) E0.5. The equivalent of E19.5 is referred to as postnatal day (P) P0.

### Genetic inducible fate mapping of gcps

WT *Atoh1-CreER*^*T2*^*/+; Tau*^*lox-STOP-lox-mGFP*-*IRES-NLSlacZ*/*+*^ mice and *Atoh1-CreER*^*T2*^*/+; Tau*^*lox-STOP-lox-mGFP*-*IRES-NLSlacZ*/*+*^*; En1*^*+/hd*^*; En2*^*ntd/ntd*^ mutants were used to fate map gcps at different time-points during development. Tamoxifen (Tm) was diluted in corn oil (50–200 μg/g of body weight) and administered by subcutaneous injection to either P2, P4, P6, P8, P10, P12, P14 or P16 pups as described [31]. The quantifications of the proportion of the ML that was labeled and of the density of labeled gcs in the IGL were performed on sections spanning ~500 μm around the midline. To test for a correlation between the proportion of the ML labeled and the density of labeled gc, we analyzed three pairs of adjacent sections (one stained for X-gal, the other immunostained for GFP) in two animals. A portion of the anterior wall and of the posterior wall was quantified in each folium. To quantify the proportion of labeled ML after Tm administration at each time-point to assess for the level of gc production, we measured the total area of the ML and the area of labeled ML using Neurolucida software, and calculated the percentage of ML that was labeled. At least 3 sections were analyzed per animal and 3 to 4 animals were analyzed per time-point.

### Histology and immunohistochemistry

All brains for fate mapping were dissected and immersion fixed in 4 % PFA for 30–45 min at 4 °C, then rinsed in PBS and transferred into 30 % sucrose at 4 °C O/N. Brains for proliferation and differentiation analyses were dissected after intracardiac perfusion with 4 % PFA, post-fixed for 2 h in 4 % PFA at 4 °C, then transferred into 30 % sucrose at 4 °C O/N. Brains for analysis of timing of fissure formation in *En1*^*+/−*^*;En2*^*−/−*^ mutants were prepared as above with an O/N post-fix at 4 °C. Brains were embedded in OCT (Tissue-Tek), frozen in methyl-butane and cryosectioned at 12 μm. Immunohistochemistry for GFP and β-Gal histochemistry were performed following standard protocols [31]. BrdU and Ki67 immunostaining required antigen retrieval by pretreatment with Na^+^ citrate buffer, (pH 6, 10 mM Sodium Citrate, 0.05 % Tween) for 10 min at room temperature, then transferred in heated Na^+^ citrate buffer for 30 min at 95 °C, cooled in Na^+^ citrate buffer and rinsed in PBS. Primary antibodies: rabbit anti-GFP (1:2000; Invitrogen), mouse anti-Ki67 (1:500; BD), sheep anti-BrdU (1:5000; Biodesign international), mouse anti-Pax6 (1:500; Covance). Secondary antibodies: biotinylated goat anti-rabbit (1:500; Vector), donkey anti-rabbit-488, anti-mouse-555 and anti-mouse-488, and goat anti-sheep-488 (all at 1:500; Invitrogen).

### Proliferation analysis-S-phase index

10 mg/ml BrdU in PBS was injected subcutaneously using a Hamilton syringe (100 μg/g) 30 min before euthanizing the mice at P2, P6, P10, P14 or P16. BrdU was incorporated in cells undergoing S-phase during the 30 min preceding euthanasia. Sections were stained for BrdU and Ki67, a marker of cycling cells. The level of proliferation was assessed as the S-phase index, or the percentage of BrdU positive; Ki67 positive cells of the total Ki67 positive population at P2, P6 and P10 in WT animals and P6 and P10 in *En1*^*+/−*^*;En2*^*−/−*^ mutants. Three animals of each genotype were analyzed and for each animal 3 sections were analyzed with an interval of 144 μm between sections. Whole lobules were analyzed at P2. For P6 and P10, after determining that there were no local differences within lobules, only a portion of the posterior wall starting at the top of the lobule was quantified. At least ~200 BrdU positive cells per section per lobule were counted. The results from the 3 sections were averaged and three animals were averaged per time point and genotype. At P14 and P16, proliferation was analyzed qualitatively, BrdU was injected 1 h before euthanasia, and sections were double stained for BrdU and Pax6 (granule cell marker) to reveal the presence of cycling cells.

### Differentiation analysis-quitting fraction

10 mg/ml BrdU in PBS was injected subcutaneously using a Hamilton syringe (100 μg/g) 24 h before euthanizing the mice at P2, P6, or P10 for WT animals and P6 and P10 for *En1*^*+/−*^*;En2*^*−/−*^ mutants and *En2*^*+/−*^ controls. Sections were stained for BrdU and Ki67. BrdU is incorporated in cells undergoing S-phase for 30 min to 1 h following the injection. After completing S-phase, cells that incorporate BrdU undergo G2 and M phases, after which they either exit the cell cycle or undergo another cell division. Thus, 24 h after BrdU injection, BrdU-labeled cells are either Ki67 negative or Ki67 positive. We chose 24 h as the time of analysis since the cell cycle length in gcps at P10 was estimated to be ~19 h [32], thus most of the cells in S-phase at the time of BrdU injection will have completed a full cell cycle. The level of differentiation was assessed by calculating the quitting fraction, which is the percentage of BrdU positive and Ki67 negative cells of the total BrdU positive population. Three sections 144 μm apart were analyzed from each of three animals of each genotype. Whole lobules were analyzed at P2, and at P6 and P10 only a portion of the posterior wall starting at the top of the folium was quantified. At least ~200 BrdU positive cells per section per lobule were counted. The results from the 3 sections were averaged and three animals were averaged per time point and genotype.

### EGL thickness measurements

The outer EGL was defined as the Ki67 positive portion of the EGL and the inner EGL as the Ki67 negative portion of the EGL. Using sagittal sections near the midline from three animals of each genotype, the area of the outer and inner EGL and length of the outer surface of each lobule were determined for three sections per animal using Neurolucida software. The thickness was calculated as the average area divided by the length.

### Microscopy

Mosaic bright-field and fluorescence images were taken on a Zeiss inverted microscope (Zeiss, Observer.Z1) using the Tile module of the Axiovision software (Zeiss). Images of the entire Cb were taken with a 10X objective, and images of individual lobules for proliferation or differentiation analyses were taken with a 40X objective.

### Quantification of the timing of fissure formation at the midline in *En1*^*+/−*^*;En2*^*−/−*^ mutants

Cerebella were collected every day between P1 and P5 (*n* = 3 or 4 at each stage), cryosectioned in series at 12 μm and every 6^th^ slide Nissl stained. Whether fissures were present at the midline was recorded. Fissures were identified based on an analysis of m-l series of sections for each mouse.

### Statistical analyses

All analyses were performed with Prism. Comparisons between lobules across different stages were done using two-way ANOVA followed by posthoc multiple comparisons tests (Tukey’s for the unrestricted inter-lobule comparisons and Dunnet’s for the comparisons using one lobule as reference). Comparisons between genotypes and lobules at individual stages (or between genotypes and lobule ratios across different stages) were done using two-way ANOVA followed by Sidak’s multiple comparisons test. The analyses were performed following a randomized block approach, to account for the matched measurements between different lobules from the same animal at each time point. All tests used α = 0.05. 3 different animals were used for each stage and each genotype. The correlation between the density of labeled gc bodies and the proportion of labeled parallel fibers (gc axons) in the ML was tested using the Spearman correlation test. The data used to determine gc production over time was fitted to third-order polynomial equations using Prism and their first derivative was generated to estimate growth rates at each time point.

## Results

### The proportion of granule cells produced during development correlates with the size of the molecular layer generated

Genetic inducible fate mapping of gcps was utilized as a means to assess the kinetics of gc production in different zones of the Cb. The gcp specific *Atoh1-CreER* mouse line [10] was combined with the *Tau*^*lox-STOP-lox-mGFP*-*IRES-NLSlacZ*^ reporter line [28] in order to mark the majority of gcps in the EGL at different time-points, and determine the proportion of marked gcs that were produced from the time of induction to the end of Cb morphogenesis (P28). With the *Tau* reporter line the cell body of differentiated gcs is visualized via expression of nuclear-localized β-galactosidase and the parallel fibers through expression of myristoylated GFP. With our experimental scheme, marked differentiated gcs in adult *Atoh1-CreER/+*; *Tau*^*lox-STOP-lox-mGFP*-*IRES-NLSlacZ/+*^ mice (referred to as *Atoh1-Tau*) are those that were produced from gcps that underwent recombination (marking) within 48 h of tamoxifen (Tm) administration. In order to determine the proportion of gcs produced during different developmental time periods, we marked gcps at 7 time points (*n* = 3 mice per time point). We expected the absolute number of gcps marked in each *Atoh1-Tau* animal to vary for each time point since 100 % recombination is not reached using inducible Cre systems, and that the efficiency of recombination might vary between time points [31]. Given that parallel fibers stack upon one another from inside to outside in the ML, we reasoned that although the proportion of marked cells could vary between *Atoh1-Tau* mice given Tm on the same day, the proportion (or height) of the ML occupied by the marked gcs that differentiated after Tm administration would be the same irrespective of the level of induction between animals. Moreover, the proportion of ML occupied by marked axons should decrease the later Tm is administered, with more and more of the inner region not being marked at late stages.

**Fig. 1:**
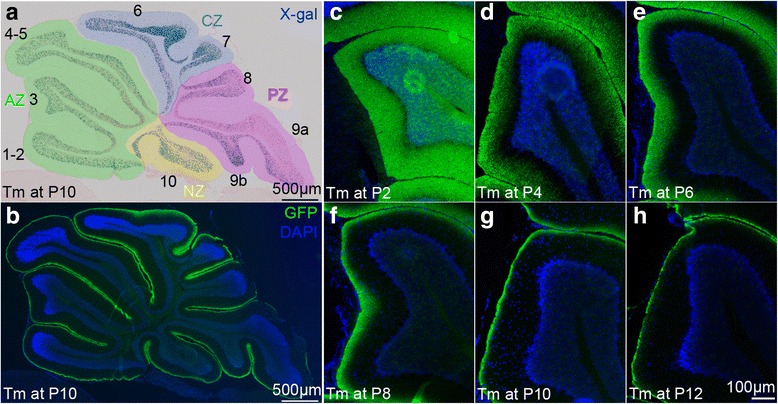
The axons of newly produced gcs stack inside to out in the molecular layer. **a**-**b** Adjacent sagittal midline sections of a P28 *Atoh1-tau* Cb after Tm administration at P10 stained for (**a**) X-gal to visualize the nuclei of the marked cells, (**b**) GFP fluorescent immunostaining (*green*) to visualize the axons of the marked granule cells. The anteroposterior zones have been color coded (anterior zone: *green*, central zone: *blue*, posterior zone: *purple* and nodular zone: *yellow*). **c**-**h** sagittal midline sections of lobule 3 of P28 *Atoh1-tau* Cb stained for GFP to reveal the position of the axons of the marked granule cells after Tm administration at the indicated ages showing that the proportion of molecular layer labeled diminishes as Tm is administered later

**Fig. 2:**
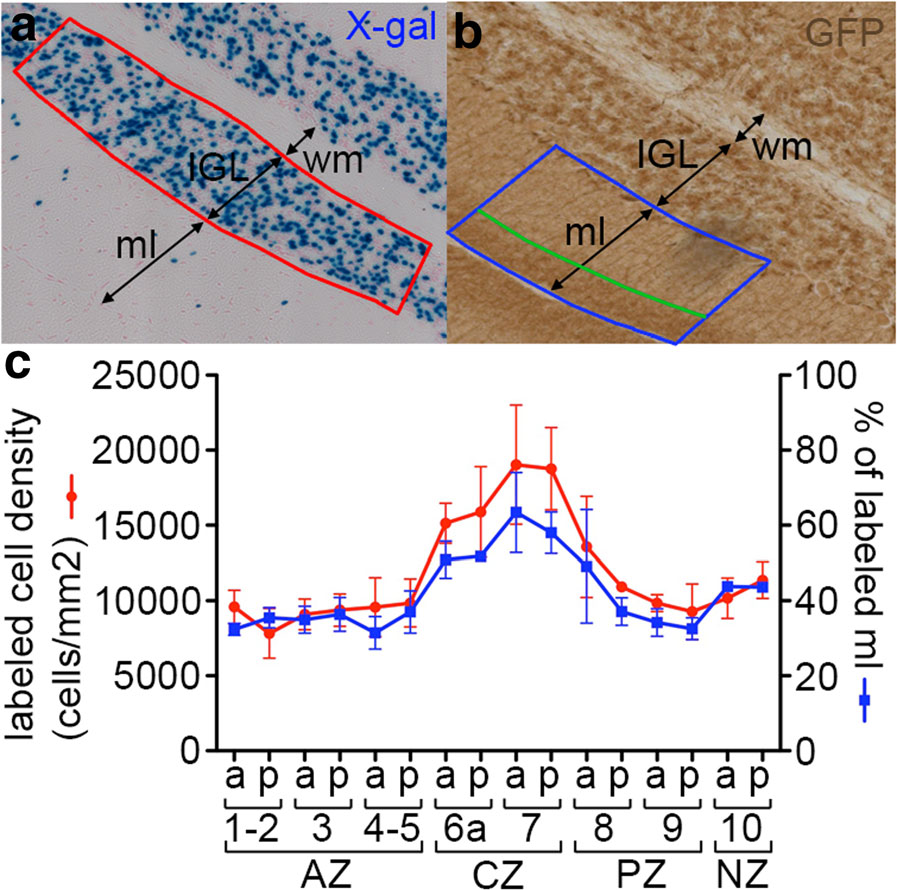
The percentage of labeled molecular layer reflects the proportion of gcs produced after tamoxifen injection. **a-b** Adjacent sagittal midline sections of a P28 *Atoh1-tau* Cb after Tm administration at P8 stained for X-gal to visualize the nuclei of marked gcs in the IGL (**a**) and for GFP DAB immunostaining to visualize the position of the axons of the marked gcs in the ml (**b**). The density of labeled nuclei was quantified in the area indicated by the red box. The % of labeled ml area was calculated as the percentage of the ml area (outlined in blue) occupied by labeled axons (outlined in *blue and green*) in a corresponding region on the adjacent section. **c** quantification of the density of labeled cell bodies (*red*) and of the % of labeled ml (*blue*) calculated in corresponding portions of the anterior (a) and posterior (p) wall of each lobule on adjacent sections such as in (**a**) and (**b**). 3 pairs of adjacent sections were quantified showing a strong correlation between the density of labeled cell bodies and the percentage of ML labeled. IGL: internal granule cell layer, ml: molecular layer, wm: white matter, AZ: anterior zone, CZ: central zone, PZ: posterior zone, NZ: nodular zone

Tm was injected every two days from P2 to P14 to *Atoh1-Tau* mice, 1 day for each mouse, and the fate-mapped cells were analyzed at P28. As expected, the cell bodies (detected with X-gal staining) of the marked cells were scattered throughout the IGL (Fig. 1a), whereas the labeled axons (immunostained for GFP) were stacked on top of unlabeled axons defining an outer portion of labeled ML and inner portion of unlabeled ML (Fig. 1b-h). We did indeed observe that the height of the outer ML that was labeled progressively decreased as Tm was administered later in Cb development (Fig. 1c-h). We measured the area of labeled ML as a proportion of the total area of ML in each lobule on three midline sections from animals that received 50 μg/g (*n* = 1) or 200 μg/g (*n* = 2) of Tm at P8. As expected, we did not observe a significant difference in the proportion of ML area labeled in each lobule between animals that received different doses of Tm, except for lobule 10 where curiously the proportion of ML area labeled was slightly greater in the 1 *Atoh1-Tau* animal induced with 50 μg/g compared to the two animals induced with 200 μg/g Tm (33.9 % compared to 29.9 % (*t*-test, *p* = 0.0015) or 30.8 % (*t*-test, *p* = 0.0045)). We then compared the density of labeled cell nuclei (Fig. 2a) to the proportion of labeled ML area (Fig. 2b) in corresponding regions of each lobule, 2 adults were injected with 50 μg/g Tm at P8 in order to distinguish individual labeled cells. We indeed found a high correlation between the density of labeled cell nuclei in particular lobules and the proportion of labeled ML area in the two animals analyzed (Spearman correlation coefficient *r* = 0.8897, *p* < 0.0001, Fig. 2c, and *r* = 0.7055, *p* = 0.0048, not shown). Thus, the proportion of labeled ML area represents the proportion of axons in the ML produced from the time of induction until the completion of gc production.

### Timing of granule cell production varies primarily between lobules of different vermal zones, with the central zone being relatively delayed

**Fig. 3:**
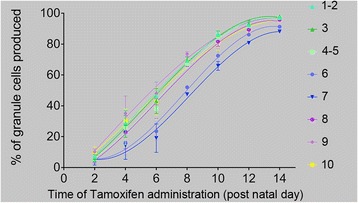
The kinetics of accumulation of gcs is distinct in each lobule from P2 to P14. Graph showing the accumulation of gcs over time in each lobule calculated as follows from the percentage of ML labeled (i.e., the % of gcs that remained to be produced) after Tm administration at the indicated times in P28 animals: % of gcs produced = 100 %–% of labeled ML (*n* = 3 animals per time-point, error bars indicate SD). A third order polynomial equation was fitted to the data; the curves have been color coded to indicate which of the four cerebellar zones the lobule is associated with (*green*: anterior zone, *blue*: central zone, *purple*: posterior zone, and *yellow*: nodular zone)

Examination of midline sections of P28 *Atoh1-Tau* mice administered Tm at P10 and stained for X-gal showed a striking difference in the density of marked gcs in the lobules corresponding to different zones, with the AZ and PZ having the least gcs marked (Fig. 1a). Consistent with this observation, quantification of the proportion of labeled ML area and density of gcs labeled at P8 revealed differences between lobules in particular zones (Fig. 2c). Given that more cells should be produced after the time of Tm administration in lobules with a higher proportion of the ML labeled, our finding raised the possibility that the timing of gc production differs more between zones than between lobules within a zone. We tested this idea by calculating the percentage of gcs that had already been produced on the day of Tm administration in *Atoh1-Tau* mice as the proportion of the ML area that was not labeled at P28 and generated a cumulative graph of gc production in each lobule (*n* = 3 or 4 mice per time point) (Fig. 3). For statistical tests (Additional file 1: Table S1A-B), we compared lobules 3, 7 and 10 as representing the AZ, CZ and NZ.

The cumulative graphs of gc production revealed that each lobule has a distinct profile of gc accumulation (Fig. 3). As predicted, the lobules within the AZ or CZ had similar profiles to each other but distinct between the two zones. Lobule 8 representing the PZ had kinetics in between the AZ and CZ, and the curve for lobule 10 was more similar to the AZ at P2-P8 and to lobule 8 at later stages. Since lobule 9 is shared between the PZ (anterior 9) and NZ (posterior 9), conclusions cannot be drawn from this lobule. When the percentage of gcs that had been produced at each two successive time points was compared in each lobule, significant difference were found for most comparisons (Additional file 1: Table S1A), showing that significant accumulation of gcs can be detected over 2 days with our fate mapping approach. Interestingly, while the percentage of gcs produced was significantly different in lobule 3 between P2 and P4 and between P4 and P6 and for lobule 10 between P2 and P4 (Additional file 1: Table S1A), neither was the case in lobule 7, indicating a delay in major accumulation of gcs at early stages in CZ lobules. Conversely, there was a significant change in accumulation of gcs in lobule 7 between P10 and P12 but not for lobules 3 and 10, supporting our conclusion that there is a general delay in gc production in lobule 7 compared to lobules 3 and 10. As predicted, we found that in lobule 7 of the CZ there was a significantly smaller percentage of gcs produced at any time point we tested (up to P14) than in lobules 3 or 10 (Fig. 3 and Additional file 1: Table S1B). Moreover, a change in gc production between P10 and P14 was significant for lobules 7 and 10, but not for lobule 3 (Additional file 1: Table S1A), demonstrating that lobules in each zone have different dynamics of gc accumulation. Finally, 11.8 % of the gcs in lobule 7 were produced after P14, whereas only 2.7 or 3.8 % in lobule 3 or 10, respectively (Fig. 3). These results reveal that maximum gc production is delayed in the lobules of the CZ compared to those of the other zones.

To further test the similarities and differences between gc accumulation in distinct lobules, the data were fitted to several mathematical models and a third-order polynomial equation was chosen as the best overall fit for all the lobules. Non-linear regression analysis was used to test the null hypothesis that the accumulation of gcs within each lobule could be fitted to the same equation, which was rejected by the test (*p* < 0.0001), emphasizing the difference in the profiles of gc accumulation between lobules. As the shape of the curves suggested that gc accumulation in lobules 6 and 7 followed a different dynamics than in other lobules (Fig. 3), we repeated the regression analysis excluding lobules 6 and 7, and found that one curve could fit all the remaining data (*p* = 0.1962). Conversely, regression analysis of gc accumulation exclusively in lobules 6 and 7 indicated that both sets of data could be fit to the same curve (*p* = 0.1812). Finally, we plotted the first derivative of the polynomial functions (i.e., the growth rate) to estimate when maximum gc production was reached for each lobule. We found this was attained later in the lobules of the CZ (~P8) than in the lobules of the other zones (~P5-P6 for most of the other lobules) (Additional file 2: Figure S1 and Additional file 1: Table S2).

### The distinct kinetics of granule cell production between zones are accompanied by different levels of proliferation and differentiation

**Fig. 4:**
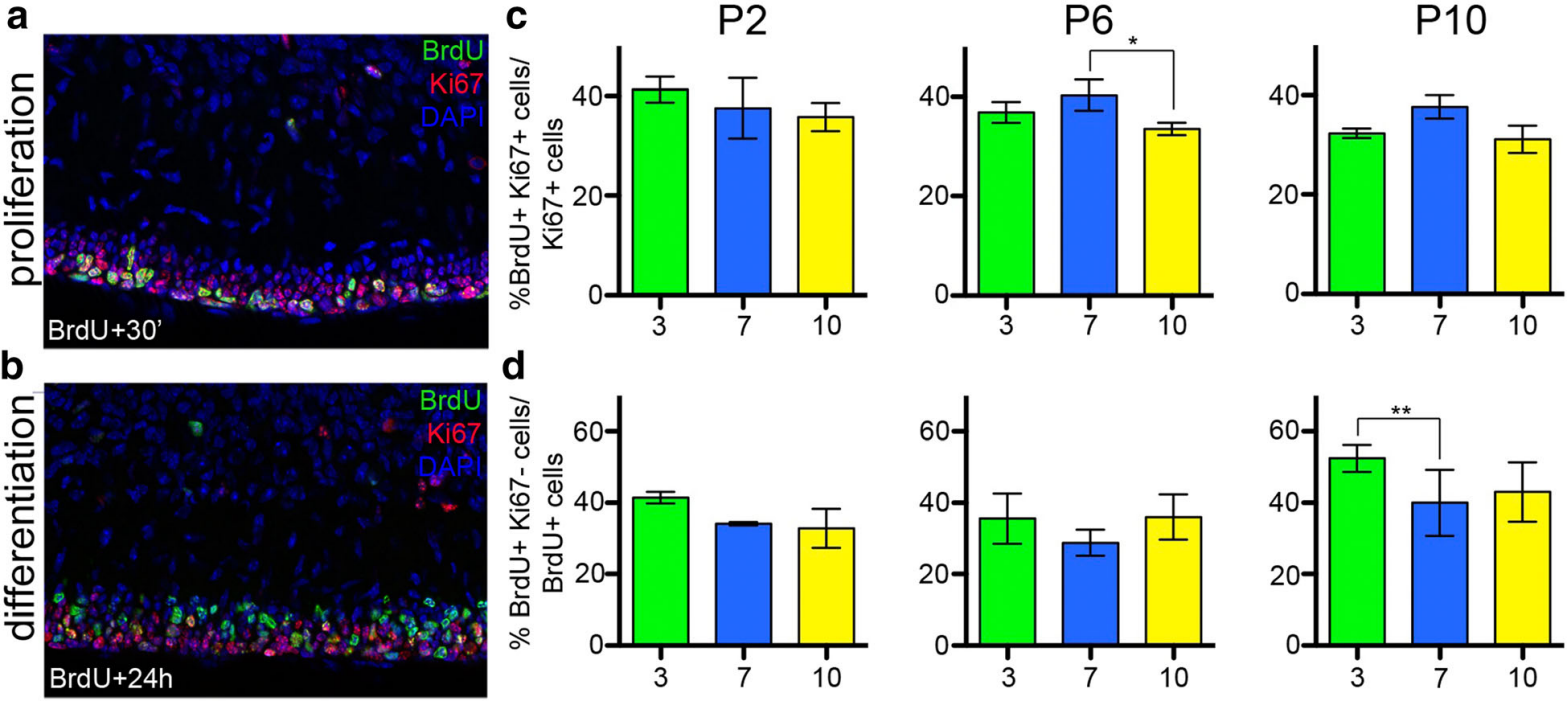
The kinetics of differentiation and proliferation are different in the anterior and central zones. **a**-**b** Portion of lobule 10 at P10 from sagittal midline sections are shown. **a** BrdU was administered 30 min before analysis marking cells in S-phase, Ki67 marks proliferating cells. **b** BrdU was administered 24 h before analysis, the cells that retain BrdU and are Ki67 negative are the cells that exited the cell cycle during the last 24 h. **c** Quantification of the level of proliferation as the percentage of Ki67 positive cells that are BrdU positive. **d** Quantification of the level of differentiation as the percentage of BrdU positive cell that are BrdU positive and Ki67 negative in the EGL. Quantifications were performed at P2, P6 and P10 in lobules 3 (AZ, *green*), 7 (CZ, *blue*) and 10 (NZ, *yellow*). *p*-values of the Dunnet’s post-hoc multiple comparisons tests following 2-way ANOVA comparing the levels of proliferation and differentiation between lobules at each time-point are shown when significant (see Additional file 1: Table S3)

Gcs are produced primarily as a result of the proliferation and expansion of the gcp pool and the timing of gc differentiation. Gcp proliferation maintains or expands the pool of precursors in the EGL, and also provides cells for differentiation. The latter eventually leads to a depletion of the pool of precursors. To test whether the delay in CZ gc production is due to less proliferation/differentiation before P6 compared to other zones and/or more proliferation/differentiation after P8, we assessed gc proliferation and differentiation in lobule 7 (CZ) compared to lobules 3 (AZ) and 10 (NZ) at P2, P6 and P10 (Fig. 4c, d). The thymidine analog BrdU was injected into mice 30 min before analysis to mark cells in S-phase and the level of proliferation (S-phase index) was calculated as the percentage of cells in S-phase (BrdU+) of all cycling gcps (Ki67+) (Fig. 4a). To assess for the level of differentiation, we determined the quitting fraction, by injecting BrdU 24 h prior to analysis and calculating the percentage of BrdU+ cells in the EGL region negative for Ki67 (Fig. 4b).

**Fig. 5:**
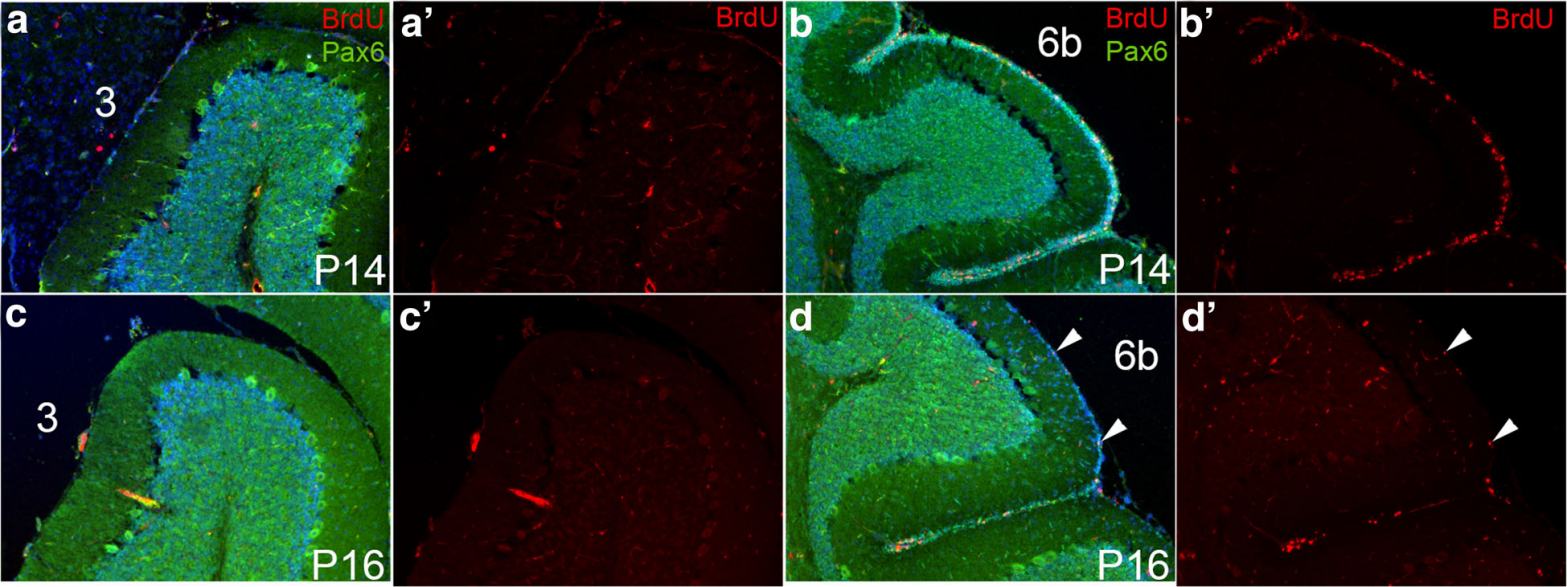
Proliferating cells persist longer in lobules of the central zone than in the anterior zone. (**a**, **b**) P14 midline sagittal sections of lobule 3 (**a**) and lobule 6b (**b**), BrdU was administered 1 h before analysis. PAX6 marks gcs in the EGL and IGL. (**a**’, **b**’) BrdU labeling alone is shown. (**c**, **d**) P16 midline sagittal sections of lobule 3 (**c**) and lobule 6b (**d**), BrdU was administered 1 h before analysis. (**c**’, **d**’) BrdU labeling alone is shown. Arrowheads indicate BrdU positive cells

At P2, we did not find significant differences in the S-phase indices between lobules 3, 7 and 10 (Fig. 4c, Additional file 1: Table S2). However, differentiation (quitting fraction) tended to be higher in lobule 3 (41.5 %) compared to 7 (34.1 %), with borderline significance (*p* = 0.0541, Fig. 4d, Additional file 1: Table S3). These results suggest differentiation rather than proliferation rates might be a more important determinant of the lower production of gcs between P2 and P4 in lobule 7. At P6, the level of differentiation was lowest in lobule 7, especially compared to lobule 10 (Fig. 4d, Additional file 1: Table S3, see also Fig. 8b). The proliferation index of gcps in lobule 7 at P6 was significantly higher than in lobule 10 (*p* = 0.0458, Fig. 4c, see also Fig. 8a), and similar to lobule 3. At P10 the level of differentiation was significantly higher in lobule 3 compared to 7 (*p* = 0.0025, see also Fig. 8b). In contrast, the level of proliferation was higher in lobule 7 than in 10 with borderline significance (*p* = 0.0540) and appeared higher than in 3 (although not significantly) (Fig. 4c, Additional file 1: Table S3, see also Fig. 8a). The results at P10 are consistent with a more active (proliferative) EGL in lobule 7 (CZ) at later time-points compared to lobules 3 and 10, and greater differentiation in lobule 3 (AZ). In addition, when a 30’ pulse of BrdU was given at P14 or P16, many BrdU labeled cells were detected in lobules 6b and 7, whereas almost no EGL cells were present in lobule 3 (Fig. 5) or 10 (not shown). Therefore the EGL remains active at P14-P16 in the lobules of the CZ, whereas the gcps are depleted in lobules of the other zones by P14. This result is consistent with our finding that gc production is almost complete at P14 in lobules of the AZ, PZ and NZ whereas more than 10 % of gcs are produced after P14 in the CZ (Fig. 3).

### Timing of gc production is least altered in the central zone of *En1*^*+/−*^*;En2*^*−/−*^ mutants

**Fig. 6:**
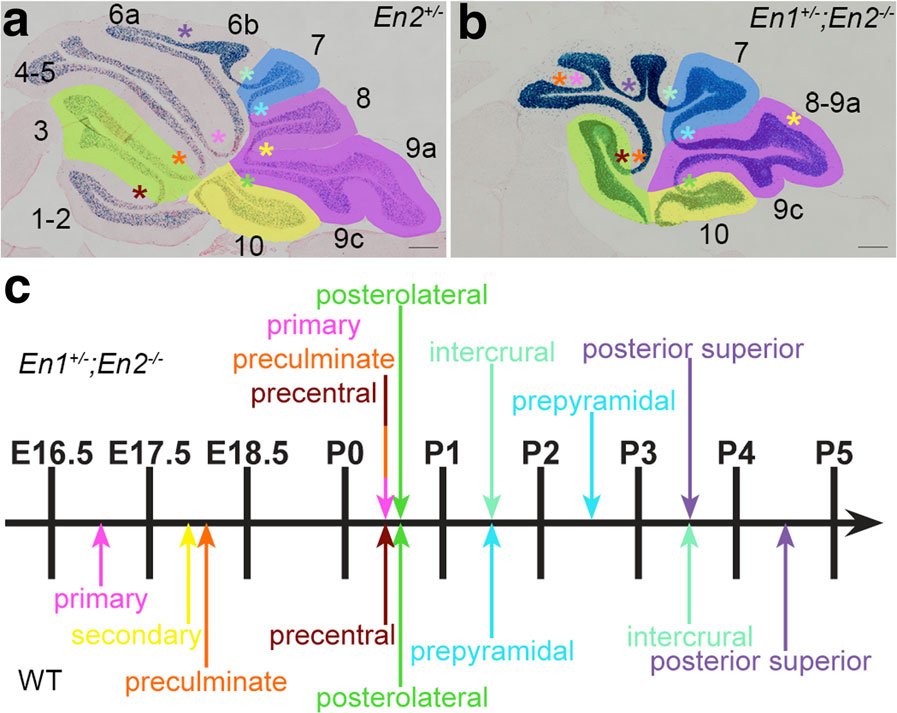
Fissures form in a different order and over a shorter time window in *En1* ^*+/−*^ *;En2* ^*−/−*^ mutants. **a** Midline sagittal section of an *En2* ^*+/−*^ control animal. **b** Midline sagittal section of an *En1* ^*+/−*^ *;En2* ^*−/−*^ mutant. Lobules in which the timing of gc production was quantified are color-coded (3 = AZ lobules in *green*, 7 = CZ lobule in *blue*, 8–9 = PZ lobules in *purple*, and 10 = NZ lobule in *yellow*). **c** Timing of formation of the fissures at the midline in controls (*bottom*) and *En1* ^*+/−*^ *;En2* ^*−/−*^ mutants (*top*). Fissures are indicated in (**a, b**) by color-coded asterisks. The morphology of the anterior lobules in the mutant is variable (one to three fissures were present anterior to the posterior superior fissure, an example with 2 fissures is shown in (**b**) and the identity of the fissures could not be unequivocally determined, therefore the detection of an anterior fissure was recorded as precentral/preculminate/primary fissure (*tricolored arrow*)

We next asked whether morphological changes in lobules are associated with significant changes in the timing of gc production by analyzing *En1*^*+/−*^*; En2*^*−/−*^ mutants that have a smaller Cb and altered foliation pattern [27], but normal basic cytoarchitecture. We chose this mutant in part because the AZ and PZ are preferentially diminished in these mutants. In most mutants, some of the fissures separating the AZ lobules are missing, and in all mutants the secondary fissure separating lobules 8 and 9 is greatly diminished resulting in a fusion of the two lobules. The CZ and NZ are comparatively spared, but nevertheless reduced in size (Fig. 6a, b). If the timing of gc production is important for 3D shape acquisition, then it should be altered preferentially in the AZ and PZ of these mutants.

Since a change in the timing of when two adjacent fissures form correlates with a change in the shape/size of the intervening lobule, we first determined when each fissure forms at the midline of *En1*^*+/−*^*;En2*^*−/−*^ mutants compared to WTs. The fissures were identified based on analyzing complete series of m-l sagittal sections [25]. We found that the fissures started to form later in the mutant than in the WTs and that the fissures formed in a different sequence and over a shorter time window (Fig. 6c). In the WT, fissures formed between E16.5 and P5, whereas they formed between P0 and P4 in the mutants (Additional file 2: Figure S2). Interestingly, in the WT the fissures separating lobules of the AZ (primary, preculminate, precentral) and PZ (secondary) formed first, followed by the fissure separating the NZ from the PZ (posterolateral), and then the fissure separating the PZ from the CZ (prepyramidal) and finally the fissures separating lobules of the CZ (intercrural and posterior-superior). In contrast, in the mutants formation of the primary and preculminate fissures of the AZ were delayed and formed concomitantly with the normally later posterolateral fissure of the NZ, whereas the intercrural and posterior superior fissures of the CZ formed earlier than normal, with the intercrural fissure forming before the normally earlier prepyramidal fissure (Fig. 6c). Finally the secondary fissure was absent in the mutants. These results show that the timing of fissure formation is profoundly altered in the *En1*^*+/−*^*;En2*^*−/−*^ mutants and not simply a general delay, with two fissures of the AZ delayed in timing and two of the CZ accelerated.

**Fig. 7:**
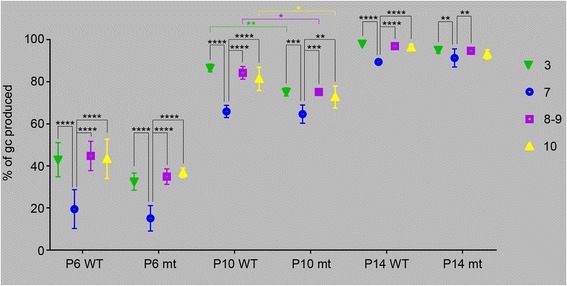
The differences in timing of gc production are attenuated between zones in *En1* ^*+/−*^ *;En2* ^*−/−*^ mutants. The percentage of gcs produced at P6, P10 and P14 in wild type (WT, same animals as in Fig. 3) and *En1* ^*+/−*^ *;En2* ^*−/−*^ mutants (mt) was calculated as in Fig. 3 (100 % - the percentage of the ML that was labeled) in lobules 3, 7, 8–9 (8 and 9 were combined in the controls since they are fused in the mutants) and 10. *p*-values of Tukey’s (for lobule comparisons) and Sidak’s (for genotype comparisons) post-hoc multiple comparisons tests following ANOVA are shown when significant (See Additional file 1: Tables S4)

We next assessed gc production in *En1*^*+/−*^*;En2*^*−/−*^ mutants carrying the *Atoh1-Tau* alleles by determining the percentage of ML area that was labeled after Tm administration at P6, P10 or P14 (stages when the differences in gc production between the CZ and other zones are large in WT)(Fig. 7) and compared the results with the measurements made previously in WT animals. Since the percentage of ML labeled is a direct measure of the portion of gcs produced after the time of marking, to assess the percentage of gcs produced at the time of marking, we used the complementary percentage of the ML that is not labeled. Because not all lobules form in mutants, we compared lobules 7 and 10 between mutant and WT, and designated the anterior-most lobule in *En1*^*+/−*^*;En2*^*−/−*^ mutants as 3 and compared it to lobule 3 in WTs, and lobules 8 and 9 were combined as one measurement since they are fused in the mutant. When gcps were marked at P6, we found that in the mutant the percentage of unlabeled ML area (gcs produced) was smaller in lobule 7 than in lobules 3, 8–9 and 10, as was the case in WTs, showing that more gcs are produced in lobule 7 after P6 and thus maximum gc production is later in the CZ than other regions of mutants as in WT mice (Fig. 7, Additional file 1: Table S4A). In addition, there were no significant difference between the percentages of gcs produced before P6 in any of the zones between mutants and WT (Additional file 1: Table S4B). In contrast, although the percentage of gcs that had been produced by P10 was lower in lobule 7 than in the other lobules, as in WTs (Fig. 7, Additional file 1: Table S4A) there was a significant decrease in the gcs produced in lobules 3, 10 and 8–9 before P10 in mutants compared to WT, whereas the difference was not significant for lobule 7 between WT and mutants (Fig. 7 and Additional file 1: Table S4B). At P14, the cumulative percentage of gcs produced was also diminished in the mutant lobules 3, 8–9 and 10, although not significantly, resulting in an attenuation of the differential delay in gc production between lobule 7 and the other lobules (Fig. 7, Additional file 1: Tables S4A-B). These results reveal that the degree of delay in gc production in lobule 7 compared to the other lobules is reduced in the mutant, especially at later stages. In order to be able to correct for multiple comparisons between different lobules, genotypes and time points, we reduced the number of variables by calculating the ratios of gc production between lobule 7 and each of the other lobules at each time point, and then tested whether the ratios were significantly different in the mutants compared to WT (Additional file 1: Table S4C). Consistent with our previous analysis, we found that the ratios were not significantly changed at P6 between the WT and mutant animals. However, we found a significant change in the ratios between lobule 7 and the other lobules at P10 and P14 (borderline significant for the ratio between 7 and 10 at P10). This shows that the extent of delay in gc production in lobule 7 compared to the other lobules is significantly reduced in the *En1*^*+/−*^*;En2*^*−/−*^ mutants compared to WTs. Since gc production appears unchanged in lobule 7 compared to WT (Additional file 1: Table S4B), the reduction in differences between lobule 7 and the others in mutants compared to WT is mainly due to a delay in gc production in lobules outside the CZ. We speculate that the delay in production of gcs in lobules of the AZ, PZ and NZ of mutants accounts for their delay in formation of fissures (foliation) in these zones.

### Proliferation, differentiation and EGL thickness are differentially affected in zones of *En1*^*+/−*^*;En2*^*−/−*^ mutants

**Fig. 8:**
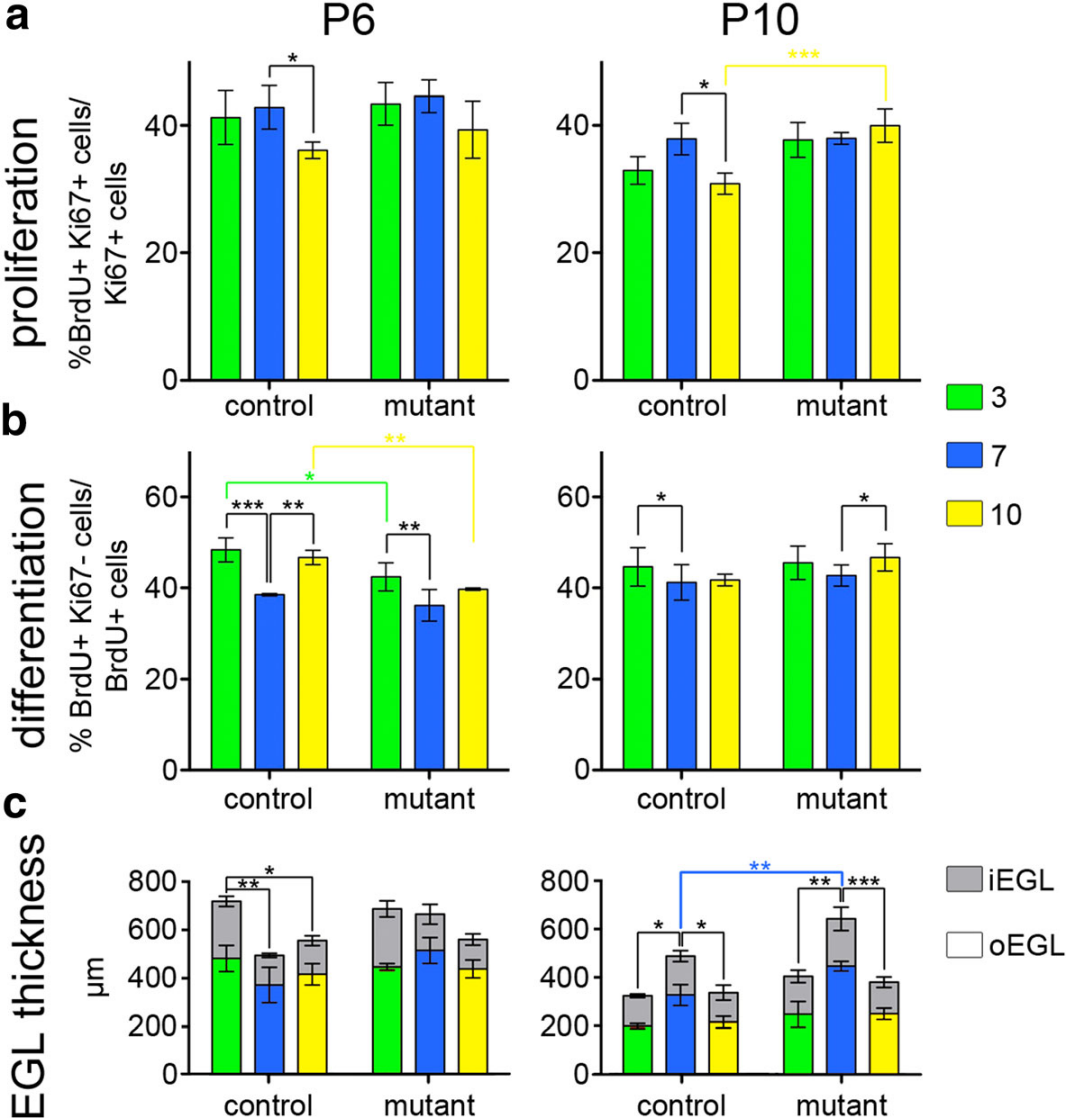
*En1* ^*+/−*^ *;En2* ^*−/−*^ mutant gcp proliferation and differentiation are altered primarily in the anterior and nodular zones. The level of gc proliferation (**a**) and differentiation (**b**) were calculated as in Fig. 4c and d, respectively, at P6 and P10 in lobules 3 (AZ: *green*), 7 (CZ: *blue*) and 10 (NZ: 10) for *En2* ^*+/−*^ control and *En1* ^*+/−*^ *;En2* ^*−/−*^ mutant mice. *p*-values of the Dunnet’s post-hoc multiple comparisons tests following ANOVA comparing the levels of proliferation and differentiation between lobules at each time-point are shown when significant (see Additional file 1: Table S5A). **c** EGL thickness in μm is shown for the same lobules at P6 and P10 (*n* = 3 mice for each measurement). The outer EGL is represented in colors and the inner EGL in *grey*. *p*-values of Tukey’s (for lobule comparisons) and Sidak’s (for genotype comparisons) post-hoc multiple comparisons tests following ANOVA are shown when significant (See Additional file 1: Tables S6)

We next tested whether the changes in the timing of gc production could be explained by changes in the rates of proliferation and/or differentiation of gcs in particular lobules of *En1*^*+/−*^*;En2*^*−/−*^ mutants compare to WTs. We quantified the proliferation index and quitting fraction of gcps in lobules 3, 7 and 10 at P6 and P10 in *En1*^*+/−*^*;En2*^*−/−*^ mutants and *En2*^*+/−*^ littermate controls (Fig. 8a, b). The *En2*^*+/−*^ animals were chosen as controls in these experiments since *En1*^*+/−*^*;En2*^*−/+*^ and *En2*^*−/−*^ animals were crossed to generate a reasonable yield of mutant animals. As expected, the *En2*^*+/−*^ animals showed similar trends as the WTs used in Fig. 4c, d, and in some cases reached additional significance, thus confirming the general trends observed using either WT or *En2*^*+/−*^ animals as controls. At both P6 and P10 we found that whereas in controls the level of proliferation in lobule 10 was significantly reduced compared to 7, there was no difference in *En1*^*+/−*^*;En2*^*−/−*^ mutants (Fig. 8a), due to an increase in the level of proliferation in the mutant lobule 10 (significant at P10) (Additional file 1: Table S5A). At both stages the proliferation indices of lobules 7 and 3 were similar between mutants and controls (Additional file 1: Table S5A). Unlike in controls where differentiation was significantly reduced in folium 7 at P6 compared to lobules 3 and 10, we observed no significant difference in the levels of differentiation between lobules 7 and 10 of *En1*^*+/−*^*;En2*^*−/−*^ mutants. The difference was still significant between lobules 7 and 3, although it appeared reduced compared to in the controls. These changes were associated with a significant reduction in differentiation in lobules 3 and 10 of mutants compared to controls (*p* = 0.023 and *p* = 0.0086, respectively; Additional file 1: Table S5A). The decrease in differentiation in mutant lobules 3 and 10 at P6 is consistent with our finding that fewer gcs are produced in these lobules compared to WTs before P10 (i.e., more gcs are produced after P10)(Fig. 7). At P10, the gcp differentiation rates were higher in lobule 3 than in 7 in the controls (Additional file 1: Table S5A), as described for WT animals (Additional file 1: Table S3). Although we found a small but significant increase in the level of differentiation in lobule 10 compared to 7 in the mutant, we did not detect any significant differences in the levels of differentiation in each lobule between controls and mutants, indicating that there are no strong differences between genotypes in each lobule at P10. Again, we turned to a ratio approach to determine whether the differences between lobules were changed in the mutant compared to WT taking into account the time variable. We found that the proliferation ratios were not significantly changed at P6, however, the ratio between proliferation in 10 and 7 was significantly changed at P10, further confirming the reduction of differences due to lower proliferation in lobule 10 of the mutant compared to WT at P10 (Fig. 7 and Additional file 1: Table S5B). Overall, we observed a leveling of proliferation and differentiation between mutant lobules compared to WT that is consistent with the more similar timing (delay) of maximum gc production between zones at all stages analyzed.

Finally, one important factor that contributes to gc production is the total number of gcps, reflected by the thickness and length of the EGL. Our previous studies revealed that the size of the Cb of *En1*^*+/−*^*;En2*^*−/−*^ mutants (and thus length of the EGL) is greatly reduced compared to WT as early as at least embryonic day 18.5 [27]. Thus, a reduction in the initial production of gcps in the embryo is likely a primary cause of the greatly reduced size of the mutant Cb. To determine whether regional difference could account for the preferential loss of the AZ compared to the CZ, we determined the thickness of the EGL (area divided by length) during post-natal development in control (*En2*^*−/+*^) and *En1*^*+/−*^*;En2*^*−/−*^ mutant mice. Correlating with the normal timing of gc production in controls, the EGL at P6 is thinner in the CZ than in other zones (Fig. 8c and Additional file 1: Table S6A) and thickest in the AZ, whereas at P10, the EGL of the CZ is the thickest (Fig. 8c and Additional file 1: Table S6B). Of significance, in mutants at P6 the thickness of the EGL of lobules 3 and 7 were similar, due to an apparent increase in the EGL thickness of lobule 7 compared to controls. At P10, the thickness of the EGL in all lobules of the mutants appeared greater than in controls, with lobule 7 being the thickest and significantly increased. The increase in EGL thickness in mutants is consistent with our fate mapping results showing prolonged gc generation in all zones, with the CZ producing the greatest proportion of gcs late.

## Discussion

To gain insight into how the diversity of cerebellar lobule shapes and sizes are generated during development, we focused on the dynamics of gc production in individual lobules as well as the four vermis zones, since gc production from proliferating precursors is crucial for the formation of lobules [33–37]. Using genetic inducible fate mapping, we first determined the timing of maximum production of gcs in individual lobules and revealed that the timing of maximum gc production in the lobules of the CZ is delayed compared to other zones. The delay in gc production in CZ lobules correlates with a thinner EGL and reduced differentiation at early postnatal stages compared to the AZ lobules and to a thicker EGL and prolonged proliferation at late stages. Furthermore, we found that in cerebella of *En1*^*+/−*^*;En2*^*−/−*^ mutants that have a preferential decrease in the sizes of lobules in the AZ and PZ the timing of gc production outside the CZ is delayed compared to normal and therefore all the zones are more similar (See model in Additional file 2: Figure S3). The delayed gc production in the mutant zones correlated with a thicker EGL and lower level of differentiation at P6 and a trend towards higher proliferation at P10. The differences in levels of differentiation and proliferation are mild, raising the question of whether other factors also contribute to the change in size and morphology of lobules in *En1*^*+/−*^*;En2*^*−/−*^ mutants. One contributor is the size of the initial pool of gcps, which is reduced in mutants, but might also be preferentially spared in the CZ of mutants. In this respect it could be relevant that we found that in the mutant the timing of formation of fissures is altered, with the fissures in the CZ (intercrural and posterior superior) actually forming earlier than in controls whereas all the others form later or not at all. There is also a much greater variability in the position of fissures in mutants compared to normal mice that can be detected at the earliest stages of foliation (Additional file 2: Figure S2). Thus, the initial proportions of the gcps allocated to each lobule is altered in the mutants compared to the WT, and this likely sets the stage for the changes in the final relative sizes of lobules in *En1*^*+/−*^*;En2*^*−/−*^ mutants. At late stages of development, the thickness of the EGL (with a thicker EGL producing more gcs) and gcp proliferation and differentiation then contribute to fine-tuning the final lobule size.

### Role of differential timing of gc production in the overall shaping of the Cb

We recently demonstrated that once the anchoring centers at the base of the fissures are formed, they act as gcp lineage restriction barriers, and as a consequence each lobule can be considered an independent developmental compartment, possibly with distinct proliferation/differentiation properties [20]. Furthermore, the cell number and geometry of gc clones is different in the two types of lobules: long lobules contain clones that are more elongated along the a-p than m-l axis and have a greater number of cells, suggesting that cell behaviors such as proliferation of precursors and the dispersion of their progeny are differentially regulated in individual lobules.

Our finding that the dynamics of gc production varies between lobules and over developmental time is consistent with our hypothesis that gc precursor behaviors can be differentially regulated in distinct lobules, reinforcing the concept that lobules act as developmental units. Our present study uncovered a higher order of regulation of gc production dynamics at the level of the zones, or groups of adjacent lobules, that are especially distinct in the AZ and CZ. Moreover, we previously found that the shape and size of gc clones is altered in the *En1*^*+/−*^*;En2*^*−/−*^ mutants, such that they resemble WT clones that are in short lobules, consistent with the *En1*^*+/−*^*;En2*^*−/−*^ mutant Cb being smaller than normal. Interestingly, all the vermis lobules are more similar in size in the *En1*^*+/−*^*;En2*^*−/−*^ mutants than in WTs, as the long lobules become shorter while the small lobules of the CZ are relatively unchanged. The thickness of the EGL is also more similar between lobules in mutants than controls at P6, as is proliferation and differentiation at P6 and P10. Interestingly, our detailed analysis of the sequential timing of formation of particular fissures in the *En1*^*+/−*^*;En2*^*−/−*^ mutant revealed that all the fissures form during a shorter period of time than normal. This result is consistent with the fact that the lobules of the AZ, PZ and NZ that are normally defined by fissures that form earlier than the fissures separating the lobules of the CZ are defined later in the mutants and at a time more similar to when the CZ lobules are defined. Thus, the timing of several developmental programs seem to be more similar between zones in *En1*^*+/−*^*;En2*^*−/−*^ mutants and this leads to the generation of lobules more similar in size than normal.

### Delay in gc production in the wild-type central zone coincides with other delayed developmental processes

We found that production of gcs and thickening of the EGL is delayed in the CZ, and a larger proportion of gcs are produced after P10 than in other zones. A major contributor to the late production of gcs is prolonged proliferation in the CZ, as the EGL persists longer in late cerebellar development (after P14). Proliferation of gcps relies on the mitogen SHH secreted by the Pcs [33, 36, 38, 39]. Interestingly, the onset of *Shh* expression in the Pcs that underlie the CZ is delayed until after birth compared to the other zones, as well the expression of *Gli1*, a transcriptional target of SHH signaling in gcps [40].

Another indication of the relative developmental delay of the CZ compared to the other zones is the delay in the maturation of Pcs located in the CZ [21]. By P5, Pcs of the CZ are organized in a monolayer but their morphology is not yet fully mature compared to Pcs located in other zones. In this context, it is interesting to note that the fissures separating the lobules within the CZ (posterior superior, intercrural) form later than the fissures separating lobules in the AZ (precentral, preculminate), raising the question of whether fissure formation is linked to gc production or EGL thickness.

The molecular mechanisms controlling the timing and positioning of fissures are not well understood. *En1* and *En2* are some of the only genes shown to play an important role in the positioning and timing of fissure formation [21, 24, 25], however, the expression pattern of *En1/2* at the time of fissure formation is not indicative of where a fissure will form [27, 41]. Instead, the *En* genes likely control processes upstream of fissure formation. Although a few genes have expression patterns restricted to a subset of lobules (*Tlx3 (or Hox11L2 or Rnx,* [42])*, Otx2* [43], *Gli1* [40], *Fgfs* [44])*,* none are restricted to a single lobule and their roles in shaping the lobules are not clear. However, *Tlx3* is mainly expressed in the CZ, *Gli1* is low in the CZ before P8, and *Otx2* is restricted mainly to the NZ, thus these genes might regulate the higher order gc production dynamics we have identified. SHH-GLI signaling is the main factor known to regulate gcp proliferation/differentiation. However, different levels of SHH have not been reported in Pcs of long and short lobules, although it is low in the early CZ [40]. Therefore, a combinatorial code of gene expression might underlie generation of distinct lobule shapes/sizes, but how it would function remains to be elucidated.

### Evo-Devo relevance, functional significance

The grouping of the lobules of the vermis into AP zones has a functional basis since lobules within a zone display a similar adult pattern of striped gene expression (e.g., ZebrinII, Hsp25 and PLCβ4 [4, 5]) and some mutants show zone-specific foliation defects ([27, 45]). Moreover mossy fiber afferents from distinct origins target particular zones and the lobules of each zone are included in distinct circuits: the NZ participates in the vestibular system, the AZ and PZ in the spino-cerebellar circuit and the CZ is preferentially connected to the neocortex via the pontine nucleus [46]. Based on appearance of each zone during evolution, the vestibulocerebellum, spinocerebellum and pontocerebellum are alternatively named the archicerebellum, paleocerebellum and neocerebellum, respectively. Thus the CZ along with the hemispheres - that comprise mostly lobules continuous with the CZ lobules - are the newest to have evolved. Moreover, the cerebellar hemispheres have expanded in parallel with areas of the neocortex during evolution [47, 48], indicating that as new functions are added, new modules are developed or existing modules expended. It is therefore interesting that we found that the growth characteristics of the CZ is the most distinct compared to other lobules in the vermis. This finding is also consistent with a modular organization of the Cb, in which each lobule can be seen as an individual module harboring a specific set of circuits that can be expanded during evolution in parallel with the distant centers it connects with. Based on our results, a-p zones can be considered as groups of modules that not only share similar inputs/outputs but also similar dynamics of development events.

## Conclusions

Our studies of WT mice reveal that gc production is differentially regulated in each of the four a-p zones of the medial vermis, and our mutant analyses highlight that the dynamics of gc production play a role in determining the 3D structure of the Cb (Additional file 2: Figure S3). In particular, the timing of gc production is specific to each lobule, most similar between lobules within a zone, and relatively delayed in the central zone lobules due to a later onset of thickening of the EGL and maximum gc production, as well as prolonged proliferation of gc progenitors. Furthermore, in *Engrailed* mutants with a smaller Cb and altered foliation pattern, gc production, proliferation and differentiation is altered such that the differences between zones are attenuated compared to WT mice, and most altered in lobules with the greatest morphological defects. Thus, the known modular organization of the Cb previously uncovered at the genetic, lineage, morphological and circuit organization levels are accompanied by modular regulation of gc production during development.

## Additional files

Additional file 1: Statistical analyses. **Table S1A**: Statistical analyses of comparisons of gc accumulation in lobules 3, 7 and 10 between 2 time-points. **Table S1B**: Statistical analyses of comparisons of gc accumulation between lobules 3, 7 and 10 at each time-point. **Table S2**. Theoretical calculation of stage for maximum gc production. **Table S3**. Statistical analysis of comparisons of the levels of proliferation and differentiation between lobules at each time-point. **Table S4A**: Statistical analysis of the comparisons of the levels of gc production between lobules at P6, P10 and P14 in WTs and in *En1* ^*-/+*^ *;En2* ^*-/-*^ mutants (mt). **Table S4B**: Statistical analysis of the comparisons of the levels of gc production in each lobule between WTs and *En1* ^*-/+*^ *;En2* ^*-/-*^ mutants. **Table S4C**: Statistical analysis of the comparisons of the ratio between gc production in lobule 7 and other lobules between WTs and *En1* ^*-/+*^ *;En2* ^*-/-*^ mutants. ﻿**Table S5A**: Statistical analysis of the comparisons, at P6 or P10, of the levels of proliferation and differentiation between lobules and between control (ctr) and *En1* ^*-/+*^ *;En2* ^*-/-*^ mutant (mt). **Table S5B**: Statistical analysis of the comparisons between genotypes of the ratios between lobule 7 and other lobules. **Table S6A**: Statistical analysis of the comparisons of EGL thickness between lobules and between control (ctr) and *En1* ^*-/+*^ *;En2* ^*-/-*^ mutant (mt) at P6. **Table S6B**: Statistical analysis of the comparisons of EGL thickness between lobules and between control (ctr) and *En1* ^*-/+*^ *;En2* ^*-/-*^ mutant (mt) at P10. (DOC 113 kb) Additional file 2:
**Figure S1**: Rate of accumulation of gcs over time in each lobule. **Figure S2**: Foliation is greatly delayed and the pattern is highly variable in *En1* ^*+/-*^ *;En2* ^*-/-*^ mutants. **Figure S3**: Model of how changes in fissure formation and production of gcs in the anterior cerebellum can alter the morphology of lobules. (PDF 648 kb)

## Abbreviations

3D: Three dimensional
a-p: Anteroposterior
AZ: Anterior zone
Bg: Bergmann glia
Cb: Cerebellum
CZ: Central zone
E: Embryonic day
EGL: External granule cell layer
Gcs: Granule cells
iEGL: Inner external granule cell layer
IGL: Inner granule cell layer
ML: Molecular layer
m-l: Mediolateral
NZ: Nodular zone
oEGL: Outer external granule cell layer
P: Postnatal day
PcL: Purkinje cell layer
PFA: Paraformaldehyde
PZ: Posterior zone
Tm: Tamoxifen
VZ: Ventricular zone
