# Supplementary material for: Differential timing of granule cell production during cerebellum development underlies generation of the foliation pattern
Source: Neural Dev. 2016 Sep 8;11(1):17. doi: 10.1186/s13064-016-0072-z (PMC5017010; doi:10.1186/s13064-016-0072-z)
Supplement: Additional file 1: — Statistical analyses. Table S1A: Statistical analyses of comparisons of gc accumulation in lobules 3, 7 and 10 between 2 time-points. Table S1B: Statistical analyses of comparisons of gc accumulation between lobules 3, 7 and 10 at each time-point. Table S2. Theoretical calculation of stage for maximum gc production. Table S3. Statistical analysis of comparisons of the levels of proliferation and differentiation between lobules at each time-point. Table S4A: Statistical analysis of the comparisons of the levels of gc production between lobules at P6, P10 and P14 in WTs and in En1 -/+ ;En2 -/- mutants (mt). Table S4B: Statistical analysis of the comparisons of the levels of gc production in each lobule between WTs and En1 -/+ ;En2 -/- mutants. Table S4C: Statistical analysis of the comparisons of the ratio between gc production in lobule 7 and other lobules between WTs and En1 -/+ ;En2 -/- mutants. ﻿Table S5A: Statistical analysis of the comparisons, at P6 or P10, of the levels of proliferation and differentiation between lobules and between control (ctr) and En1 -/+ ;En2 -/- mutant (mt). Table S5B: Statistical analysis of the comparisons between genotypes of the ratios between lobule 7 and other lobules. Table S6A: Statistical analysis of the comparisons of EGL thickness between lobules and between control (ctr) and En1 -/+ ;En2 -/- mutant (mt) at P6. Table S6B: Statistical analysis of the comparisons of EGL thickness between lobules and between control (ctr) and En1 -/+ ;En2 -/- mutant (mt) at P10. (DOC 113 kb) [file 13064_2016_72_MOESM1_ESM.doc]

**Supplementary Tables**

**Tables S1: Statistical analysis of gc accumulation over time.**

2-way ANOVA detected significant difference between lobules (p<0.0001) and between stages (p<0.0001) with a significant interaction between the two variables (p<0.0001).

|  | **P2 vs. P4** | **P4 vs. P6** | **P6 vs. P8** | **P8 vs. P10** | **P10 vs. P12** | **P12 vs. P14** | **P10 *vs.* P14** |
| --- | --- | --- | --- | --- | --- | --- | --- |
| **3** | **<0.0001****** | **0.0135*** | **<0.0001****** | **0.0038**** | 0.6712 | 0.9178 | 0.1132 |
| **7** | 0.2048 | 0.9095 | **<0.0001****** | **0.0011**** | **0.0140*** | 0.5553 | **<0.0001****** |
| **10** | **0.0002***** | 0.0608 | **<0.0001****** | **0.0464*** | 0.5418 | 0.5974 | **0.0152*** |

**Table S1A: Statistical analyses of comparisons of gc accumulation in lobules 3, 7 and 10 between 2 time-points.**

Comparisons between time-points in individual lobules were done using Tukey’s post-hoc multiple comparisons test, adjusted p-values are shown, significant p-values are bold.

(Related to Figure 3)

|  | **3 vs. 7** | **7 vs. 10** | **3 vs. 10** |
| --- | --- | --- | --- |
| **P2** | 0.3878 | **0.0031**** | 0.0730 |
| **P4** | **<0.0001****** | **<0.0001****** | 0.3322 |
| **P6** | **<0.0001****** | **<0.0001****** | 0.9756 |
| **P8** | **<0.0001****** | **<0.0001****** | 0.6136 |
| **P10** | **<0.0001****** | **0.0015**** | **0.0038**** |
| **P12** | **<0.0001****** | **<0.0001****** | **0.0156*** |
| **P14** | **<0.0001****** | **<0.0001****** | 0.4351 |

**Table S1B: Statistical analyses of comparisons of gc accumulation between lobules 3, 7 and 10 at each time-point.**

Comparisons between lobules at each time-point were done using Tukey’s post-hoc multiple comparisons test, adjusted p-values are shown, significant p-values are bold.

(Related to Figure 3)

|  | **1-2** | **3** | **4-5** | **6** | **7** | **8** | **9** | **10** |
| --- | --- | --- | --- | --- | --- | --- | --- | --- |
| **B2** | 0.6870 | 0.9555 | 1.406 | 2.636 | 2.489 | 1.192 | 0.1984 | 0.5287 |
| **B3** | -0.04758 | -0.05818 | -0.07422 | -0.1119 | -0.1016 | -0.06488 | -0.02769 | -0.03671 |
| **Stage of m gc pr**  **(post-natal day)** | 4.8 | 5.5 | 6.3 | 7.9 | 8.2 | 6.1 | 2.4 | 4.8 |

**Table S2. Theoretical calculation of stage for maximum gc production**

The stage at which maximum growth is attained was calculated using the second derivative of the equations fitted to the data shown in Figure 3. Maximum gc production is reached when the second derivative, ie. the slope of the growth rate curve is equal to 0. The data have been fitted to a third order polynomial equation

f(x)= **B3**x^3^+**B2**x^2^+**B1** where f(x) represents gc production as a function of time.

Its first derivative is f’(x)= 3**B3**x^2^+2**B2**x and its second derivative is f’’(x)= 6**B3x**+2**B2**

Thus the maximum of granule cell production is reached when f’’(x)=0 ie. x=-2**B2**/6**B3**.

The Table shows the B2 and B3 parameters of the equation fitted to each lobule gc production and the calculated stage of maximum gc production (in post-natal day).

|  | **P2** | | **P6** | | **P10** | |
| --- | --- | --- | --- | --- | --- | --- |
|  | proliferation | differentiation | proliferation | differentiation | proliferation | differentiation |
| **3 vs. 7** | 0.3081 | **0.0541** | 0.3565 | 0.076 | 0.1182 | **0.0025**** |
| **7 vs. 10** | 0.7332 | 0.8843 | **0.0458*** | **0.0596** | **0.054** | 0.5135 |

**Table S3: Statistical analysis of comparisons of the levels of proliferation and differentiation between lobules at each time-point.**

2-way ANOVA detected a significant difference between lobules (p=0.0194) and time-points (p=0.0195) with no significant interaction between the two variables across the proliferation data. 2-way ANOVA detected a significant difference between lobules (p=0.0008) but not between time-points (p=0.0634) with no significant interaction between the two variables across the differentiation data.

Adjusted p-values shown in the table correspond to Dunnet’s multiple comparisons test that used lobule 7 as a reference.

(Related to Figure 4)

**Tables S4: Statistical analysis of gc production in WT and *En* mutants.**

At each time-point, 2-way ANOVA was performed. At P6, a significant difference was detected between lobules (p<0.0001) but not between genotypes (p=0.2122), with no significant interaction between the two variables (p=0.0868). At P10, a significant difference was detected between lobules (p<0.0001) and between genotypes (p=0.0357) with a significant interaction between the two variables (p=0.0106). At P14, a significant difference was detected between lobules (p<0.0001) but not between genotypes (p=0.3402) with a significant interaction between the two variables (p=0.0030).

|  | **3 vs. 7** | **7 vs. 10** | **3 vs. 10** | **3 vs. 8-9** | **7 vs. 8-9** | **10 vs. 8-9** |
| --- | --- | --- | --- | --- | --- | --- |
| **P6 WT** | **<0.0001****** | **<0.0001****** | >0.9999 | 0.8910 | **<0.0001****** | 0.9618 |
| **P6 mt** | **<0.0001****** | **<0.0001****** | 0.1688 | 0.7135 | **<0.0001****** | 0.8926 |
| **P10 WT** | **<0.0001****** | **<0.0001****** | 0.0607 | 0.5854 | **<0.0001****** | 0.4323 |
| **P10 mt** | **0.0004***** | **0.0038**** | 0.5557 | >0.9999 | **0.0004***** | 0.5673 |
| **P14 WT** | **<0.0001****** | **<0.0001****** | 0.2059 | 0.5327 | **<0.0001****** | 0.8857 |
| **P14 mt** | **0.0026**** | 0.2122 | 0.0934 | 0.9398 | **0.0067**** | 0.2295 |

**Table S4A: Statistical analysis of the comparisons of the levels of gc production between lobules at P6, P10 and P14 in WTs and in *En1^-/+^;En2^-/-^*****mutants (mt).**

Adjusted p-values shown in the table correspond to Tukey’s multiple comparisons test between lobules.

(Related to Figure 7)

|  | **3** | **7** | **10** | **8-9** |
| --- | --- | --- | --- | --- |
| **P6 WT vs. mt** | 0.2695 | 0.8966 | 0.6714 | 0.3175 |
| **P10 WT vs. mt** | **0.0048**** | 0.9881 | **0.0349*** | **0.0255*** |
| **P14 WT vs. mt** | 0.3544 | 0.7390 | 0.2405 | 0.3544 |

**Table S4B: Statistical analysis of the comparisons of the levels of gc production in each lobule between WTs and *En1^-/+^;En2^-/-^*****mutants.**

Adjusted p-values shown in the table correspond to Sidak’s multiple comparisons test between genotypes.

(Related to Figure 7)

|  |  | **P6** | | **P10** | **P14** | |
| --- | --- | --- | --- | --- | --- | --- |
| **7:3** | WT vs. mt | 0.2165 | **<0.0001****** | | **<0.0001****** |  |
| **7:8-9** | WT vs. mt | 0.4992 | **0.0045**** | | **0.0002***** |  |
| **7:10** | WT vs. mt | 0.9376 | **0.0526** | | **0.0002***** |  |

**Table S4C: Statistical analysis of the comparisons of the ratio between gc production in lobule 7 and other lobules between WTs and *En1^-/+^;En2^-/-^*****mutants.**

2-way ANOVA was performed separately for each ratio. A significant difference was detected between genotypes (p=0.0006) and time-points (p<0.0001) in the ratio between gc production in lobules 3 and 7, with a significant interaction between the two variables (p=0.0028). A significant difference was detected between genotypes (p=0.0021) and time-points (p=0.0012) in the 7:8-9 ratio of gc production, with a significant interaction between the two variables (p=0.0397). A significant difference was detected between genotypes (p=0.0099) but not between time-points (p=0.1065) in the 7:10 ratio of gc production, with a significant interaction between the two variables (p=0.0078). Adjusted p-values shown in the table correspond to Sidak’s multiple comparisons test between genotypes.

(Related to Figure 7)

|  |  | **P6** | | **P10** | |
| --- | --- | --- | --- | --- | --- |
|  |  | proliferation | differentiation | proliferation | differentiation |
| **control** | 3 vs. 7 | 0.7256 | **0.0003***** | 0.0698 | **0.0474*** |
|  | 7 vs. 10 | **0.0355*** | **0.001**** | **0.0149*** | 0.8941 |
| **mutant** | 3 vs. 7 | 0.8312 | **0.0047**** | 0.9913 | 0.1025 |
|  | 7 vs. 10 | 0.0941 | 0.0698 | 0.5187 | **0.0239*** |
| **3** | ctr vs. mt | 0.8365 | **0.0230*** | 0.0601 | 0.9830 |
| **7** | ctr vs. mt | 0.9034 | 0.5438 | >0.9999 | 0.9248 |
| **10** | ctr vs. mt | 0.6009 | **0.0086**** | **0.0008***** | 0.2334 |

**Table S5A: Statistical analysis of the comparisons, at P6 or P10, of the levels of proliferation and differentiation between lobules and between control (ctr) and *En1^-/+^;En2^-/-^*****mutant (mt).**

At each time-point, 2-way ANOVA was performed separately for the proliferation and the differentiation data. At P6, a significant difference in proliferation was detected between lobules (p=0.0158) but not between genotypes (p=0.3021), with no significant interaction between the two variables (p=0.8959). A significant difference in differentiation was found between lobules (p=0.0001) and between genotypes (p=0.0240) with no significant interaction between the two variables. At P10, we did not detect a significant difference in proliferation between lobules (p=0.1837), but did detect a significant difference between genotypes (p=0.0033), with a significant interaction between the two variables (p=0.0377). A significant difference in differentiation was detected between lobules (p=0.0187) but not between genotypes (p=0.3718), with no interaction between the two variables (p=0.0973). Adjusted p-values shown in the table correspond to Dunnet’s multiple comparisons test for the lobule comparisons (3 or 10 vs. 7) and Sidak’s multiple comparisons test for the genotype comparisons. Although the 2-way ANOVA did not detect a significant difference in proliferation between lobules at P10, the post-hoc test resulted in a significant difference between lobule 7 and 10. In this case, there was a significant interaction between lobule identity and genotype, which suggests that the difference is significant based on the significant interaction effect. This is consistent with the results obtained in the WT (see Table S3) and thus likely to be biologically relevant.

(Related to Figure 8)

|  |  | **P6** | | **P10** | |
| --- | --- | --- | --- | --- | --- |
|  |  | proliferation | differentiation | proliferation | differentiation |
| **3:7** | ctr vs. mt | 0.9902 | 0.2540 | 0.2139 | 0.9124 |
| **10:7** | ctr vs. mt | 0.8873 | 0.1945 | **0.0253*** | 0.4096 |

**Table S5B: Statistical analysis of the comparisons between genotypes of the ratios between lobule 7 and other lobules.**

For each ratio, 2-way ANOVA was performed separately for the proliferation and the differentiation. No statistical difference was detected between genotypes (p=0.5700) or between time-points (p=0.1711) in the ratio between proliferation in 3 and 7, with no significant interaction between the two variables (p=0.3717). No statistical difference between genotypes (p=0.2472) but a statistically significant difference between time-points (p=0.0107) was detected in the ratio between differentiation in 3 and 7, with no interaction between the two variables (p=0.3886). A significant difference was detected between genotypes (p=0.0394) but not between time-points (p=0.2822) in the ratio between proliferation in 10 and 7, with no significant interaction between the two variables (p=0.1602). No significant difference was detected between genotypes (p=0.7261) or time-points (p=0.0595) in the ration between differentiation in 10 and 7, with no significant interaction between the two variables (p=0.0833).

Adjusted p-values shown in the table correspond to Sidak’s multiple comparisons test.

(Related to Figure 8)

|  |  | **oEGL** | **iEGL** | **all EGL** |
| --- | --- | --- | --- | --- |
| **control** | 3 vs. 7 | 0.0825 | **0.0007***** | **0.0070**** |
|  | 7 vs. 10 | 0.5905 | 0.6501 | 0.5067 |
|  | 3 vs. 10 | 0.3387 | **0.0021**** | **0.0356*** |
| **mutant** | 3 vs. 7 | 0.3203 | **0.0034**** | 0.9073 |
|  | 7 vs. 10 | 0.2471 | 0.3344 | 0.1778 |
|  | 3 vs. 10 | 0.9787 | **0.0006***** | 0.0968 |
| **3** | ctr vs. mt | 0.7822 | 0.9982 | 0.9010 |
| **7** | ctr vs. mt | 0.0123* | 0.8162 | 0.0160* |
| **10** | ctr vs. mt | 0.9349 | 0.5406 | 0.9997 |

**Table S6A: Statistical analysis of the comparisons of EGL thickness between lobules and between control (ctr) and *En1^-/+^;En2^-/-^*****mutant (mt) at P6.**

For oEGL, iEGL and all EGL, 2-way ANOVA was performed. In the EGL, a significant difference between lobules was detected (p=0.0096) but not between genotypes (p=0.1393) with no significant interaction between the two variables (p=0.0575). In the oEGL, no significant difference was detected between lobules or genotypes (p=0.5116 and p=0.0832, respectively) with no significant interaction between the two variables (p=0.0537). In the iEGL, a significant difference was detected between lobules (p<0.0001) but not between genotyopes (p=0.7889) with no significant interaction between the two variables (p=0.2856). Adjusted p-values were calculated using ANOVA followed by Tukey’s multiple comparisons test for the lobule comparisons and Sidak’s multiple comparisons test for the genotype comparisons. In both the EGL and oEGL, the differences in lobule 7 between the ctr and the mt appeared significant based on the post-hoc test, however, since the ANOVA did not detect a significant genotype effect in these two data sets, with no interaction between lobule identity and genotype, the results of the post-hoc tests are statistically irrelevant.

(Related to Figure 8)

|  |  | **oEGL** | **iEGL** | **all EGL** |
| --- | --- | --- | --- | --- |
| **control** | 3 vs. 7 | **0.0039**** | 0.3433 | **0.0113*** |
|  | 7 vs. 10 | **0.0087**** | 0.2805 | **0.0174*** |
|  | 3 vs. 10 | 0.8207 | 0.9865 | 0.9506 |
| **mutant** | 3 vs. 7 | **0.0002***** | 0.2869 | **0.0012**** |
|  | 7 vs. 10 | **0.0002***** | 0.0607 | **0.0006***** |
|  | 3 vs. 10 | 0.9969 | 0.5453 | 0.8394 |
| **3** | ctr vs. mt | 0.2227 | 0.4931 | 0.2201 |
| **7** | ctr vs. mt | **0.0018**** | 0.4119 | **0.0100*** |
| **10** | ctr vs. mt | 0.4854 | 0.9712 | 0.6715 |

**Table S6B: Statistical analysis of the comparisons of EGL thickness between lobules and between control (ctr) and *En1^-/+^;En2^-/-^*****mutant (mt) at P10.**

For oEGL, iEGL and all EGL, 2-way ANOVA was performed. In the EGL, a significant difference between lobules was detected (p=0.0002) and between genotypes (p=0.0213) with no significant interaction between the two variables (p=0.2241). In the oEGL, a significant difference was detected between lobules and genotypes (p<0.0001 and p=0.0083, respectively) with no significant interaction between the two variables (p=0.1183). In the iEGL, a significant difference was detected between lobules (p=0.0376) but not between genotyopes (p=0.1283) with no significant interaction between the two variables (p=0.7209). Adjusted p-values shown in the table were obtained by Tukey’s multiple comparison’s test for the lobule comparisons and Sidak’s multiple comparisons test for the genotype comparisons.

(Related to Figure 8)
