# Supplementary material for: Differential timing of granule cell production during cerebellum development underlies generation of the foliation pattern
Source: Neural Dev. 2016 Sep 8;11(1):17. doi: 10.1186/s13064-016-0072-z (PMC5017010; doi:10.1186/s13064-016-0072-z)
Supplement: Additional file 2: — Figure S1: Rate of accumulation of gcs over time in each lobule. Figure S2: Foliation is greatly delayed and the pattern is highly variable in En1 +/- ;En2 -/- mutants. Figure S3: Model of how changes in fissure formation and production of gcs in the anterior cerebellum can alter the morphology of lobules. (PDF 648 kb) [file 13064_2016_72_MOESM2_ESM.pdf]

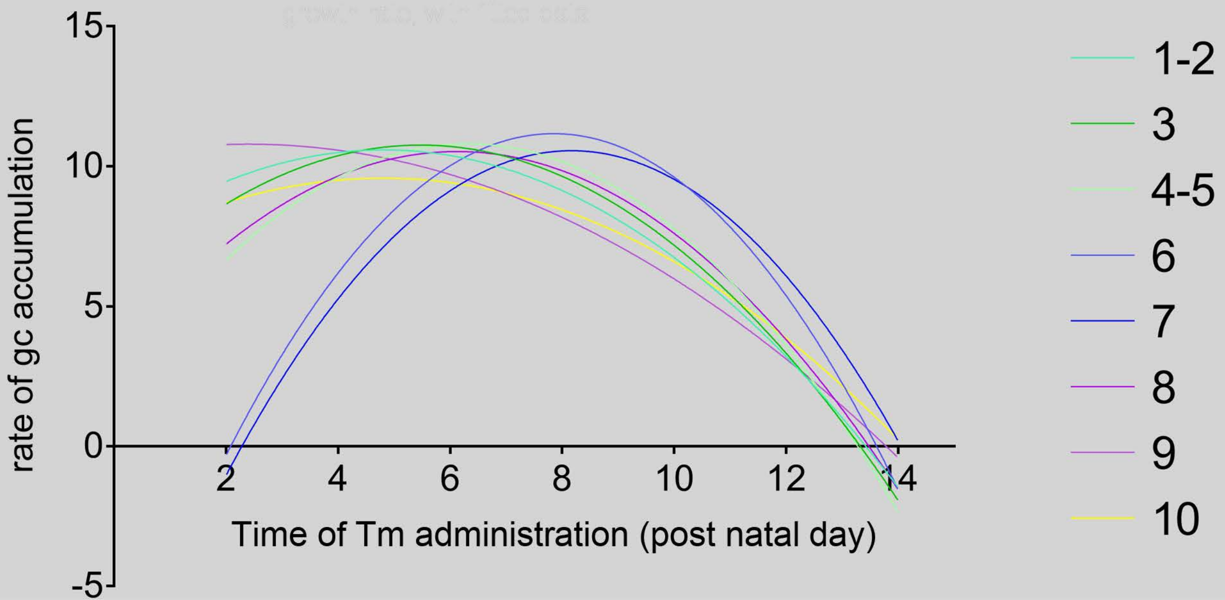

15
10
5
0
-5
rate of gc accumulation
2
4
6
8
10
12
14
Time of Tm administration (post natal day)
1-2
3
4-5
6
7
8
9
10

Figure S1

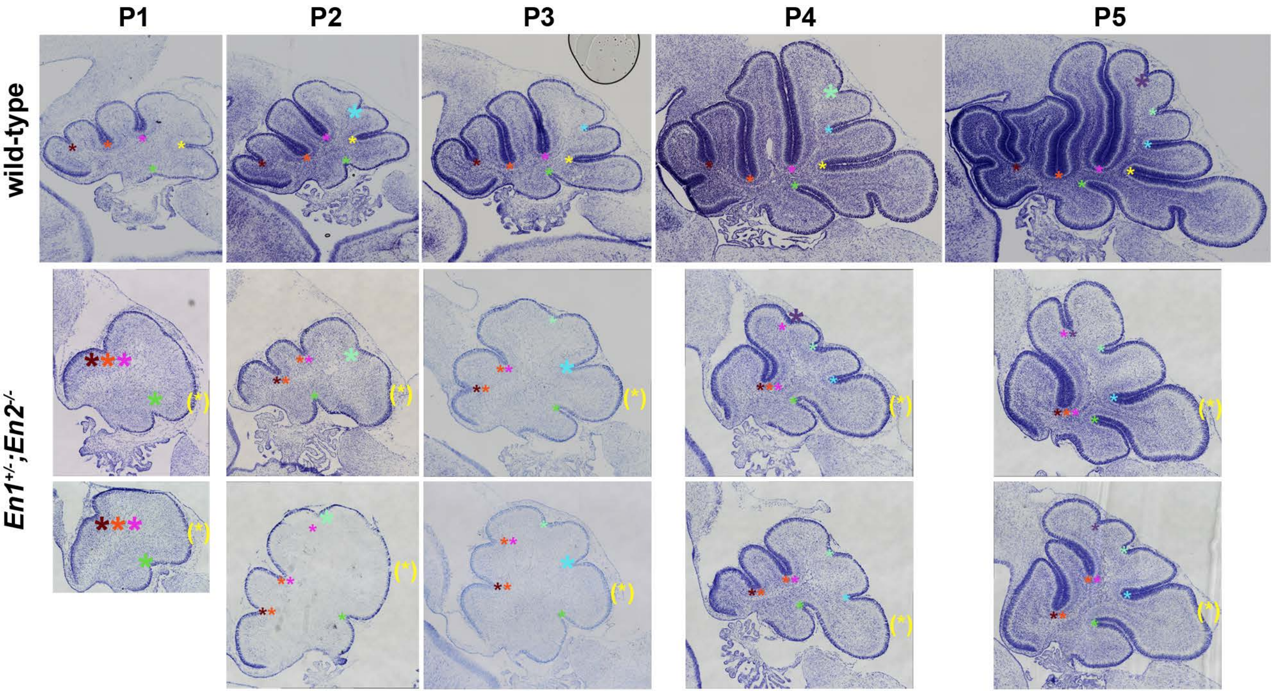

Figure S2

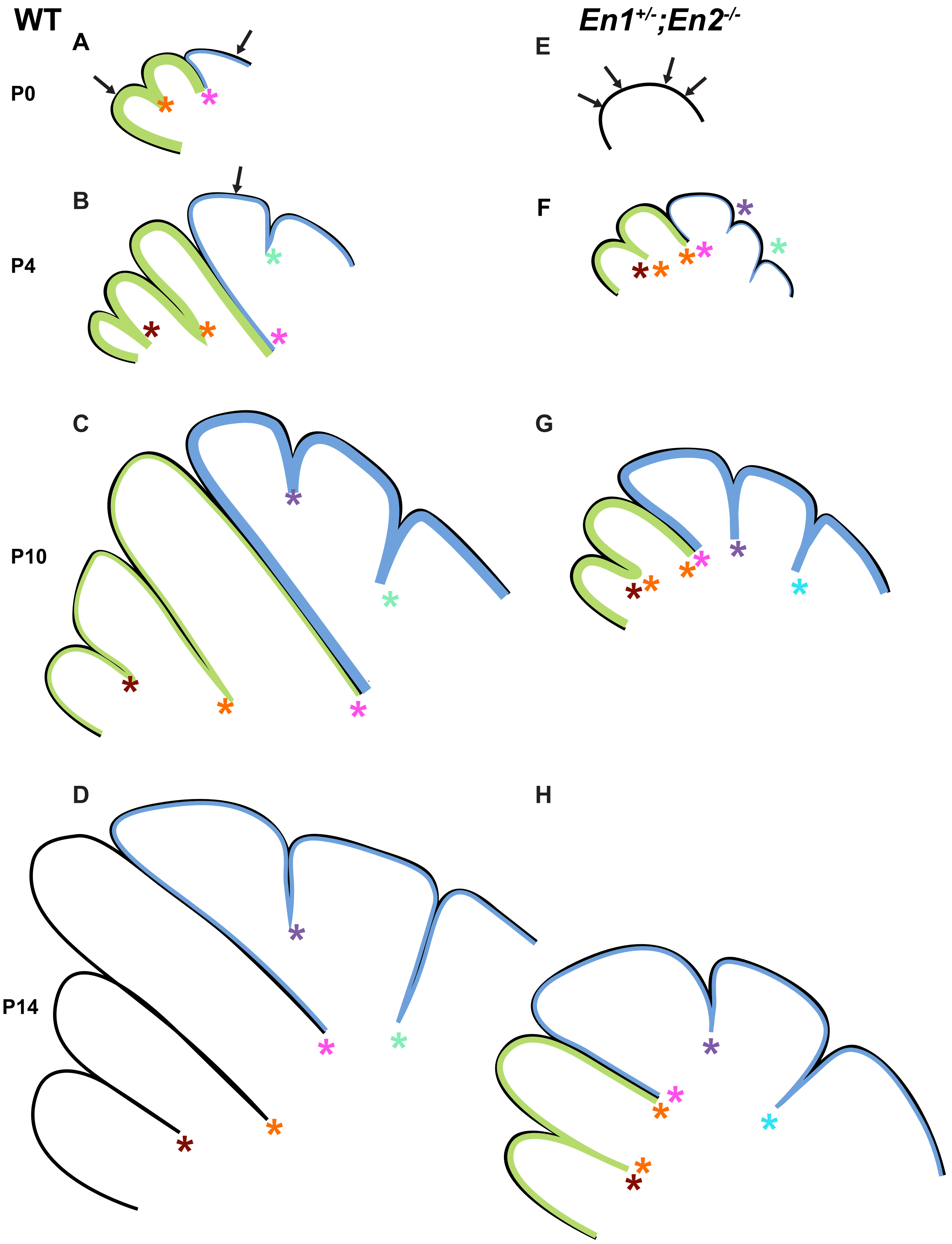

Figure S3

Supplementary Figure legends

Figure S1: Rate of accumulation of gcs over time in each lobule.
First derivative of the equations fitted to the data in Figure 3. The maximum of each derivative determines the time of maximum level of gc production for each lobule. The same color as in Figure 3 was used to identify the lobules.
(Related to Figure 3)

Figure S2. Foliation is greatly delayed and the pattern is highly variable in *En1$^{+/-}$;En2$^{-/-}$* mutants. Examples of midline sections from two *En1$^{+/-}$;En2$^{-/-}$* mutants and a WT mouse are shown at P1 to P5. Each fissure is indicated by a different color asterisk, using the color code in Figure 6. For the mutant where it is not possible to determine the exact fissures in some regions, the likely set of fissures that could be present are shown. The approximate position of the secondary fissure (yellow) in the mutant is shown in brackets. Lobule 8 is shifted posterior and fused to the anterior region of lobule 9 in mutants.

Figure S3. Model of how changes in fissure formation and production of gcs in the anterior cerebellum can alter the morphology of lobules. Schematic representations depict the differential timing of development of the AZ (green) and CZ (blue) in WT **(A-D)** and *En1$^{+/-}$;En2$^{-/-}$* mutants **(E-H)** illustrating how changes in the timing of formation of fissures and gc production can produce different morphologies. The thickness of the colored lines indicates the level of gc production at each stage (thicker = greater). The arrows indicate where fissures form between stages. As described in the results, higher granule cell production is accompanied by an increase in the thickness of the EGL and gc differentiation rate. The CZ also maintains proliferation to a later stage than the AZ, and the length of the mutant EGL is shorter than WT. **(A)** In WT at P0, 2 fissures (primary, asterisk in pink and preculminate, asterisk in orange) have already formed in the anterior Cb, between P0 and P4, two fissures are added (arrows) and gc production is greater (thicker colored line) in the AZ than in the CZ. **(B)** Higher gc production in the AZ is conserved at P4. Precentral (asterisk in brown) and intercrural (asterisk in blue) fissures are now present. Between P4 and P10, one fissure is added to the CZ (arrow). **(C)** At P10, gc production is greater in the CZ and slows down in the AZ and the posterior superior (asterisk in purple) fissure has formed. **(D)** By P14, gc production is over in the AZ while some gcs are still produced in the CZ. **(E)** In the mutant, no fissures have formed by P0. Four fissures are added to the anterior Cb between P0 and P4 (arrows). **(F)** By P4, four fissures have formed (indicated by asterisk, two colors indicate identity of fissure is not clear). Gc production is greater in the CZ compared to the AZ, but decreased in the CZ compared to WT. **(G)** At P10, gc production is greater in the CZ but gc production in the AZ is greater than in the WT. **(H)** By P14, there is still gc production in the CZ as well as in the AZ. The attenuation of the differences in gc production and timing of fissure formation contributes to the generation of distinct foliation patterns between WT and mutant.
